# Influence of social support and rearing behavior on psychosocial health in left-behind children

**DOI:** 10.1186/s12955-017-0592-1

**Published:** 2017-01-19

**Authors:** Haiyan Xing, Wei Yu, Fengjiao Xu, Sanmei Chen

**Affiliations:** 1 Department of Nursing, School of Medicine, Shaoxing University, No.900 Chengnan Avenue, Shaoxing, 312000 Zhejiang Province China; 2 Institute of Epidemiology, Shaoxing Keqiao District Center for Disease Control and Prevention, Shaoxing, Zhejiang Province China

**Keywords:** Psychological health, Social support, Rearing behavior, Left-behind children

## Abstract

**Background:**

The purpose of this study is to examine psychological health of left-behind children (LBC), social support and rearing behavior towards LBC as well as their correlations in the city of Shaoxing, China.

**Methods:**

By stratified sampling, 401 LBC and 527 non-left-behind children (NLBC) had completed the questionnaires in 2014. Spearman’s correlation was performed to clarify the relationship between psychological health, social support and rearing behavior in LBC. Multiple linear regression analytical methods were used to identify the variables that were associated with psychological health.

**Results:**

Compared to NLBC, LBC got lower scores in psychological health, general social support, subjective support and emotional warmth, but higher in rejection. Psychological health was positively correlated with social support, and negatively with rearing behavior (rejection, overprotection) in LBC. It was also closely connected with the subjective support, rejection and general health status.

**Conclusion:**

These data show that LBC suffer significant impairment on psychological health, and receive less social support and worse rearing behavior than NLBC. Psychological health may be affected by subjective support, rejection, and general health status. Urgent government assessment and support from the community, school, mental health systems are warranted.

## Background

As a result of rapid economic development and urbanization over the last three decades in China, surging numbers of rural-to-urban migrant workers are a unique phenomenon [1]. The nationwide migrant population has reached 245 million at the end of 2013, more than 1/6 of the total population, based on the 2014 Report on China’s Migrant Population Development [2]. Due to limitation of income level, housing conditions, urban educational policy and so on, many migrants are prone to leave their children in the countryside under the care of relatives and friends. These children are usually called “left-behind children” (LBC). The report released on May 2013 by China Women’s Federation indicated that over 61 million LBC in Chinese rural area, accounted for 21.88% of all national children, the increase number reached 2.42 million during five years [3].

Compared to non-left-behind children, due to their parents’ absence, LBC encountered more difficulties such as education interruption, nutrition deficiency and psychological confusion, and showed more psychological problems, less pro-social behaviors, greater communication difficulties like sensitivity, low self-esteem, proneness to violations and failure to comply disciplinary [4–6]. Shaoxing city, which locates in eastern China, has attracted a large number of migrant workers, especially in developed towns (Maan, Qianqing et al.). On the other hand, some local farmers migrate to the bigger developed cities such as Shanghai, Kunming, Hangzhou and the like, especially from underdeveloped towns (Jidong, Wangtan et al.), looking for higher family income.

Liu et al. [7] discovered that the positive rate of LBC’s psychological problems was significantly higher than that of NLBC’s. A large-scale study based on 8627 rural pupils chosen from 10 provinces showed that children living with parents had the best academic performances and LBC with only one of the parents working out performed the worst, especially those who only lived with father [8]. Huang et al. [9] found that 3 groups ranging from low to high mental health level were LBC. Stay-at-home experience cannot only influence the mental health of LBC but also continue to influence that of children who had been once left at home.

Most of these investigations suggest that the main influential factors to mental health included family relationship, family education, social network, personality trait, coping style and so on [10–12]. Previous studies reported that there were significant positive correlation between social support and mental health in LBC [7, 10, 11, 13]. According to the evidence literature shows, the parenting style reported by the subject is an important pathogenic factor, albeit non-specific, influencing individual vulnerability to mental illness [14–17].

In this study, psychosocial health of LBC was assessed by comparing with NLBC. Whether social support and rearing behavior affect psychosocial health of LBC was analyzed by investigation.

## Methods

### Subjects and procedure

Data were obtained from cross-sectional survey in 2014. The target population comprised LBC and NLBC from Chinese primary and secondary school adolescents aged 10–18, randomly selected by stratified and cluster sampling technique. At first, three stratifications were divided by economic level in Keqiao district of Shaoxing city (4, 8, 4 towns or streets respectively). Over 80% of left behind children lived in the third stratification towns, including Jidong, Wangtan, Pingshui and Xialv, so we randomly chose two of them, Wangtan and Xialv to survey. Then two primary schools and secondary schools were selected from each town, and two classes of students per grade, from grades four to nine (Since the questionnaire should be completed independently), were drawn in every school, including subsamples classified as LBC and NLBC.

For study purpose, LBC were defined as children who stayed at home with extended family members when their parents or one parent relocated elsewhere to work for at least 6 months. The control group in this study comprised NLBC, whose parents worked and lived in the same rural area. In each class, all of the students were recruited on the same day into the study. We got 928 valid questionnaires with response rate 99.3%.

### Measure of psychosocial health, social support and rearing behavior

Psychosocial health was measured by The Pediatric Quality of Life Inventory Version 4.0 (PedsQL™4.0) Generic Core Scales. The PedsQL™4.0 was translated to Chinese and validated previously in China [18], consist of 23 items in four domains: physical health (eight items), emotional functioning (five items), social functioning (five items) and school functioning (five items) [19]. The physical health summary score comprised of the physical function scale, and the psychosocial health summary score comprised of the emotional, social, and school functioning scales [20]. The internal consistency reliability for Total Scale Score (Cronbach’s a = 0.90),Physical Health Summary Score (a = 0.81),and Psychosocial Health Summary Score(a = 0.89) were excellent [18]. The psychosocial health summary score represent the level of psychosocial health. A higher score indicates better psychosocial health.

Social support was measured by the Social Support Rating Scale (SSRS). The SSRS was first reported by Xiao in 1994, had been confirmed to have good reliability (Cronbach’s a = 0.89, test-retest reliability = 0.92) and validity and was appropriate for the Chinese population [21, 22], consisting of 10 items in three domains: objective support, subjective support, and support utilization. The general support score is the total score from the three domains. A higher score represents more social support [21].

Short-Egna Minnen av Barndoms Uppfostran Chinese version (s-EMBU-c) was used to measure rearing behaviors. The s-EMBU-c consists of 23 items in three domains: rejection, emotional warmth and overprotection, which was developed from the original 81-items version [23, 24]. Indices of reliability were between 0.74 ~ 0.84 for the internal consistency (Cronbach’s a coefficient), 0.73 ~ 0.84 for split-half reliability,0.70 ~ 0.8 l for test-retest estimates. Confirmative factor analysis found the scale had good construct validity [23]. Because parents of left-behind children did not live with them for a long time, guardian here could be a father, mother, grandfather, grandmother, or even others.

### Statistical analysis

Statistical analyses performed by SPSS version 18.0 software, included the *χ* ^*2*^ test for sociodemographic characteristics and independent-samples *t*-tests for age, psychosocial health, SSRS and s-EMBU-c scores. Spearman’s correlation was performed to identify the relationship between psychosocial health, social support and rearing behavior in LBC. Multiple linear stepwise regression was performed to assess the impact of related variables. Variables in the model included gender, age, one or both parent(s) going out, the time with parents out, academic record, general health status, three domains of SSRS and three domains of s-EMBU-c. The variance inflation factor (VIF) of all variables was less than 1.1 in final model based on collinearity diagnositics. Academic record, general health status (bad, medium and good) and the time with parents out (less than one month, one month to three months and more than three months) were classified into three levels, so two dummy variables were established respectively. Variables such as gender, age, one or both parent(s) going out, the time with parents out, academic record, general health status (medium vs. bad), objective support, support utilization, emotional warmth and overprotection were excluded by stepwise regression.

## Results

### Sociodemographic characteristics

Data were obtained from 401 left-behind children and 527 non-left-behind children. Their sociodemographic characteristics are shown in Table 1. No significant differences in age, gender, grade, academic record and relations with classmates were found between groups. Father, mother and parents accounted for 68.7%, 3.8% and 27.4% respectively in the farmers leaving home, 51.0% of whom came back from every one week to three months.

**Table 1:** Sociodemographic characteristics of left-behind children and non-left-behind children

		LBC n (%)	NLBC n(%)	*P* value
*n*		401	527	
Age (years; mean ± standard deviation)		14.2 ± 1.8	14.2 ± 1.9	0.812
Gender	Boy	193(48.1)	273(51.8)	0.268
	Girl	208(51.9)	254(48.2)	
Grade	Primary school 7–12 years	139(34.7)	178(33.8)	0.778
	Middle school 13–15 years	262 (65.3)	349(66.2)	
Academic record	Good	127(41.4)	180(58.6)	0.550
	Medium	128(45.6)	153(54.4)	
	Bad	118(42.0)	163(58.0)	
Relations with classmates	Well	261(41.8)	364(58.2)	0.269
	Normal	127(46.2)	148(53.8)	
	Bad	8(57.1)	6(42.9)	

### Psychosocial health, social support and rearing behavior between the two groups

Psychological health of LBC was lower than NLBC’s (*P* < 0.05; Table 2).

**Table 2:** Distribution of psychosocial health,SSRS and s-EMBU-c scores in left-behind children and non-left-behind (mean ± standard deviation)

	LBC	NLBC	*P* value
PedsQL™4.0			
Psychosocial health	79.60 ± 13.02	81.26 ± 12.36	0.048
SSRS			
General support	43.19 ± 6.74	44.18 ± 0.44	0.021
Subjective support	25.67 ± 4.13	26.62 ± 3.75	<0.001
Objective support	9.49 ± 2.65	9.50 ± 2.60	0.951
Support utilization	8.03 ± 2.03	8.06 ± 2.04	0.837
s-EMBU-c			
Rejection	1.49 ± 0.44	1.44 ± 0.43	0.042
Emotional warmth	2.52 ± 0.63	2.60 ± 0.66	0.042
Overprotection	2.03 ± 0.49	1.99 ± 0.45	0.222

No significant differences in objective support and support utilization were found between the two groups. However, LBC’s general support and subjective support were lower (*P* < 0.05 for each comparison; Table 2).

There’s no significant difference in overprotection. However, LBC’s rejection was higher and emotional warmth was lower than NLBC’s (*P* < 0.05 for each comparison; Table 2).

### Psychosocial health and influential factors in left-behind children

When the convergent validity between the psychosocial health, social support and rearing behavior was analyzed in LBC, most correlation coefficients were significant except for emotional warmth. There was positive correlation between psychosocial health and social support, conversely, negative correlation between psychosocial health and rejection or overprotection. The psychosocial health was mostly reflected by the SSRS’s general and subjective domains. The data are reported in Table 3.

**Table 3:** Correlation coefficients among psychosocial health, social support and rearing behavior in left-behind children

	Psychosocial health	*P* value
SSRS		
General support	0.303	<0.001
Subjective support	0.312	<0.001
Objective support	0.117	0.019
Support utilization	0.217	<0.001
s-EMBU-c		
Rejection	−0.260	<0.001
Emotional warmth	0.043	0.388
Overprotection	−0.198	<0.001

Multiple linear stepwise regression analyses were used to identify variables that were associated with psychosocial health. The results showed that psychosocial health was related to subjective support, rejection, and general health status (good vs. bad) in LBC. Psychosocial health was positively influenced by subjective support and good general health status, negatively influenced by rejection (Table 4).

**Table 4:** Variables associated with psychosocial health in left-behind children, revealed by multiple linear stepwise regression

Variable	*B*	*Beta*	*t*	*P*	95%*CI* for *B*	*VIF*
Constant	64.527		12.524	<0.001	54.391 ~ 74.664	
Subjective support	0.734	0.235	4.505	<0.001	0.413 ~ 1.054	1.093
Rejection	−6.669	−0.224	−4.398	<0.001	−9.652 ~ −3.686	1.036
General health status (control = bad)						
Good	7.338	0.176	3.406	0.001	3.099 ~ 11.577	1.070

*B* unstandardized coefficients; *Beta*: standardized coefficients

*VIF* variance inflation factor; Multiple Correlation Coefficient, *R* = 0.429

## Discussion

Children are highly dependent on social welfare, and they need access to education, basic health care, entertainment and family care, which are indispensable to their growth. Due to limitation of city policy, the cost of raising children of migrants has greatly increased in the city. On the other hand, the situation of migrants themselves is very hard-- low wages and long working hours, which hampers to foster their children in the city. So they have to leave their children at home [25]. Left-behind children phenomenon is the result of large-scale labor migration between the rural and urban social system. Because of the growth environment has been systemic damage or defects in physical and mental health, learning and socialization, etc., LBC are faced with many problems [26]. Childhood is not only the first stage of life development, but also the most critical period of rapid physical and mental development. Due to the absence of parents, other guardian such as grandfather, grandmother, uncle, auntie and so on, often paid more attention to physical health (personal safety, food, clothes et al.) rather than changes on their mood and emotion. No opportunity to communicate with their families deeply affected LBC’s psychological health. LBC were often lack of security sense and had poor interpersonal communication skills [26, 27]. There is some evidence that the living situation of the Chinese LBC is getting worse [27–29] and these children are experiencing significant mental health problems, such as anxiety and behavioral problems [5, 28, 30, 31].

Our results indicated that LBC’s psychological health was much lower compared to NLBC’s. This finding corresponded to the results reported by similar studies [4, 9, 32, 33]. Jia et al. found that LBC reported poorer health-related quality of life than NLBC due to psychological dysfunction [4]. Compared with NLBC, LBC had great impairment in related factors of psychological health such as social support and rearing behavior. The scores of general support and subjective support of LBC were significantly lower than those of NLBC’s, which was similar with other studies [34, 35]. The guardian of LBC adopt less active forms,giving less emotional warmth,more likely to use the severely negative way like rejection or punishment compared to the guardian of NLBC.

A study showed that the detection rate of psychological problem was high in LBC, reached 57.14% based on the Middle School Students’ Mental Health Scale, there were significant differences in some psychological domains between gender, guardian (mother or father or others) and the time of whose parents had been out [36]. The positive rate of LBC’s psychological problems was 81.8%, significantly higher than that of NLBC’s (18.2%, P < 0.05) [25].

The present results revealed the main factor that influenced psychological health was subjective support. A positive correlation was found between the subjective support and psychological (Table 3), which was similar with other study [35, 37]. The effect of subjective support ranked first in the included variables (Table 4). The subjective support score of LBC with both parents going out was significant lower than that of those with only one parent going out (24.87 ± 4.27 vs. 26.06 ± 4.04, t = 2.556, p = 0.011). Social support impose certain effect on psychological health of left-behind children,it should attract more social concerns [7]. Social support is a function of social relationships provided by members within a social network, which is generally related to the number of or contact frequency with family members, relatives, friends, and classmates. High socioeconomic status families are capable to provide a large number of ancillary conditions and social support, such as adequate food and nutrition, parental care and attention, good learning conditions. However children of low socioeconomic status are deficient on these resources. LBC are a typical low socioeconomic status in the social life in China today [31]. Due to the distance with parents, it is more difficult for LBC to seek help. Although schools and society pay more attention to them than before, LBC still get relatively little concern and limited social support, so we should take efforts to provide enough support.

Another factor that might have contributed to the differences in psychological health was rearing behavior. Rearing behavior from father, mother or other watchers in the family system had impacts on the level of mental health for LBC [9]. In the early childhood mental development stage, parents are the most important roles to help children establish a correct outlook of judgment and value of right and wrong. Due to the lack of parental care and family love, LBC will inevitably lose their balance when they feel the differences between NLBC. Sometimes aggressive behavior will take place in order to keep the balance of their psychology [38]. The other guardians will be responsible for more things because of parents’ absence. Except for taking care of children, they also do farm work, care for the elderly losing self-care ability, so that they are exhausted and vulnerable to scold and punish the children rather than encouragement and praise. Moreover, such as inter-generational care, which grandparents take, is always a sense of difficulty, after all, the rural elderly in low educational level and backward concept, always pay more attention to adaptation rather than education, material rather than mental. So under the rearing model, it is easy for children to form excessive self or timid psychological characteristics [9]. One study found that, there was significant difference of mental health between LBC and NLBC, mainly in three aspects including anxiety, allergic tendencies and impulses propensity. In other words, the experience of staying at home influenced children’s mental health in the future [9].

Psychological health was also affected by general health status (Table 4). Compared to bad general health status, psychological health level of self-reported good general health status is higher in LBC. General health status has a positive effect on psychological health. One study showed that mental health and physical health were closely related, often sick people, who were prone to anger, liked to blame someone else. However healthy people were able to maintain a good mood, a healthy attitude, and a better social adaptability [39].

In brief psychological health of LBC was worse than NLBC’s, weak social support and traditional rearing behavior further aggravating their psychological health problem. Parents, guardian, school teachers and community workers should keep in good touch, pay close attention to the LBC’s physical and mental health. Except for encouraging LBC maintain telephone or internet contact with parents weekly or daily, the support network of various forms also should be established, including family, school and community, to jointly promote healthy growth of LBC.

### Limitations

The study only sampled two towns in this city, although it had a large sample size, the results were not applicable to all aspects and could not be generalized to whole left-behind children in China. It was difficult to establish cause and effect relationship between psychological health and influential factors based on a cross sectional study. Other factors such as disease and sudden positive or negative events that were known as influencing factors on psychological health, were not measured in this research.

## Conclusions

The findings of this study highlighted the differences of psychological health, social support and rearing behavior between the left-behind children and non-left-behind children. This analysis provided additional evidence supporting that psychological health, general social support, subjective support and emotional warmth in LBC were lower than that in NLBC, but the score of rejection were higher compared to NLBC’s. A positive correlation was found between psychological health and social support, as well as a negative correlation between psychological health and rearing behavior (rejection, overprotection). The main influential factors on psychological health were subjective support, rejection and general health status.

## Abbreviations

LBC: Left-behind children
NLBC: Non-left-behind children
PedsQL™4.0: The Pediatric Quality of Life Inventory Version 4.0
s-EMBU-c: Short-Egna Minnen av Barndoms Uppfostran Chinese version
SSRS: Social support rating scale
VIF: Variance inflation factor

## References

[1] Lu CH, Wang PX, Lei YX, Luo ZC (2014). Influence of health-related quality of life on health service utilization in Chinese rural-to-urban female migrant workers. Health Qual Life Outcomes.

[2] Department of Services and Management of Migrant Population, National Health and Family Planning Commission of China. Report on China’s Migrant Population Development. China Popul Publishing House. 2014. p. 1–3.

[3] China Women’s Federation. Report on Chinese rural left-behind children and rural-to-urban migrant children Research. Retrieved May 18, 2013, from http://news.china.com.cn/txt/2013-05/18/content_28862083.htm. Accessed 10 Dec 2015.

[4] Jia ZB, Shi LZ, Cao Y, Delancey J, Tian WH (2010). Health-related quality of life of “left-behind children”: a cross-sectional survey in rural China. Qual Life Res.

[5] Fan F, Su LY, Gill MK, Birmaher B (2010). Emotional and behavioral problems of Chinese left-behind children: a preliminary study. Soc Psychiatry Psychiatr Epidemiol.

[6] Yu XM (2011). Concerned on the vulnerability of physical and psychosocial adaptation in migrant children and left-behind children (in Chinese). Chin J Child Health Care.

[7] Liu P, Yang TH, Wei J, Zheng QN (2015). Relevant study on left-behind children’s psychological health and social support (in Chinese). J Guiyang Med Coll.

[8] Yao JH, Mao YQ (2008). Rural left-behind children’s academic psychology in Western China and the school management countermeasures. Front Educ China.

[9] Huang YP, Li L (2012). Influence of Family Education Style on Mental Health of Stay-at-home Children (in Chinese). Health Med Res Pract.

[10] Yang TH, Wei J, Liu P, Zhang SH, Zhen QN, He F (2016). Left- behind children’ mental health: the influences of personality traits and social support (in Chinese). Chin J Health Psychol.

[11] Luo GM (2014). Research on the relationship between social support, Coping Style and Mental Health among rural children left behind (in Chinese). Ment Health Educ.

[12] Lin JX, Zhao YF, Liu ZJ, Zhen BT (1999). Influence of rural family education on psychological health of children and adolescents (in Chinese). J Chin Sch Health.

[13] Chen YH, Qi JL, Zhang YB (2014). The characteristics of mental health and social support of rural left-behind children in middle school (in Chinese). J Qiqihar Univ Med.

[14] Perris C (1988). A theoretical framework for linking the experience of dysfunctional parental rearing attitudes with manifest psychopathology. Acta Psychiatr Scand Suppl.

[15] Reiss D, Hetherington EM, Plomin R, Howe GW, Simmens SJ, Henderson SH, O´Connor TJ, Bussell DA, Anderson ER, Law T (1995). Genetic questions in environmental studies. Differential parenting and psychopathology in adolescence. Arch Gen Psychiatry.

[16] Enns MW, Cox BJ, Clara I (2002). Parental bonding and adult psychopathology: results from the US national comorbidity survey. Psychol Med.

[17] Jacques JZ, Martin RE, Marcelo PF (2008). Is parental rearing an associated factor of quality of life in adulthood?. Qual Life Res.

[18] Chen YM, He LP, Mai JC, Hao YT, Xiong LH, Chen WQ, Wu JN (2008). Validity and reliability of Pediatric Quality of Life Inventory Version 4.0 generic core scales in Chinese children and adolescents (in Chinese). Chin J Epidemiol.

[19] Doostfatemeh M, Ayatollahi SMT, Jafari P (2015). Testing parent dyad interchangeability in the parent proxy-report of PedsQL™4.0: a differential item functioning analysis. Qual Life Res.

[20] Varni JW, Seid M, Knight TS, Uzark K, Szer IS (2002). The PedsQL™4.0 generic core scales: sensitivity, responsiveness, and impact on clinical decision-making. J Behav Med.

[21] Xiao SY (1994). The theoretical basis and research applications of “Social Support Rating Scale” (in Chinese). J Clin Psychiatry.

[22] Liu JW, Li FY, Lian YL (2008). Investigation of reliability and validity of the social support scale (in Chinese). J Xinjiang Med Univ.

[23] Jiang J, Lu ZR, Jiang BJ, Xu Y (2010). Revision of the Short-form Egna Minnen av Barndoms Uppfostran for Chinese (in Chinese). Psychol Dev Educ.

[24] Arrindell WA, Sanavio E, Aguilar G, Sica C, Hatzichristou C, Eisemann M, Recinos LA, Gaszner P, Peter M, Battagliese G, Kallai J, Ende JVD (1999). The development of a short form of the EMBU: Its appraisal with students in Greece, Guatemala, Hungary and Italy. Personal Individ Differ.

[25] Duan CR, Yang G (2009). Several important issues concerning on migrant children and left-behind children (in Chinese). Chin Teach.

[26] Wu F, Yang WW (2011). Defects and reconstruction of growth environment for left-behind children and migrant children: from the perspective of resilience theory (in Chinese). Popul Res.

[27] Task Force (Wu N, et al.). A survey report on the education of hometown-remaining children in rural areas (in Chinese). Educ Res. 2004,(10):15–18,53.

[28] Fan F, Sang B (2005). Absence of parental upbringing and Liushou Children’s personality, academic achievements as well as behavior problems. Psychol Sci.

[29] Li ZH, Yin XY, Zhu CY (2013). Emotional and behavioral problems of rural left-behind children: The average tendency and individual differences (in Chinese). J Hunan Agricult Univ (Soc Sci).

[30] Chi XX (2005). An analysis on the moral development problems of unattended children from a psychosocial perspective (in Chinese). Teach Educ Res.

[31] Zhou ZK, Sun XJ, Liu Y, Zhou DM. Psychological development and education problems of children left in rural areas (in Chinese). J Beijing Norm Univ (Soci Sci Ed). 2005;(1):71–9.

[32] Wang FS, Sun YH, Niu JJ, Gong L, Cai B, Sun LN (2010). SCL-90 test result on the left-behind children in rural area: a meta-analysis (in Chinese). J Hyg Res.

[33] Luo J, Wang W, Gao WB (2009). Review of the Studies On Rural Left-Behind Children in China (in Chinese). Adv Psychol Sci.

[34] Liu ZY, Zhang C (2015). Study on the effect of social support on self-awareness of left-behind children (in Chinese). Chin J Child Health Care.

[35] Qiu DP, Dai SH, Liu X (2015). Survey on negative life events and social support in left-behind children (in Chinese). Jiangsu J Prev Med.

[36] Hu K, Ding HY, Meng H (2010). Research on status of mental health in the rural left-behind children (in Chinese). Chin J Health Psychol.

[37] Gao WB, Wang Y, Wang WZ, Liu ZK (2007). Social support and interpersonal relationships in school of children left in rural areas (in Chinese). Chin Ment Health J.

[38] Meng AY (2008). Effect of different rearing style on psychological, moral growth in left-behind children (in Chinese). J Yunnan Finance Econ Univ.

[39] Zhao YM (2008). The relationship between psychology and physiology and the affection on health (in Chinese). Chin Med J Metallurg Ind.

